# Supplementary material for: Protective Mechanism of Eurotium amstelodami from Fuzhuan Brick Tea against Colitis and Gut-Derived Liver Injury Induced by Dextran Sulfate Sodium in C57BL/6 Mice
Source: Nutrients. 2024 Apr 16;16(8):1178. doi: 10.3390/nu16081178 (PMC11054642; doi:10.3390/nu16081178)
Supplement: Supplementary file 1 [file nutrients-16-01178-s001.zip › nutrients-2899652-supplementary.pdf]

Supplementary Materials

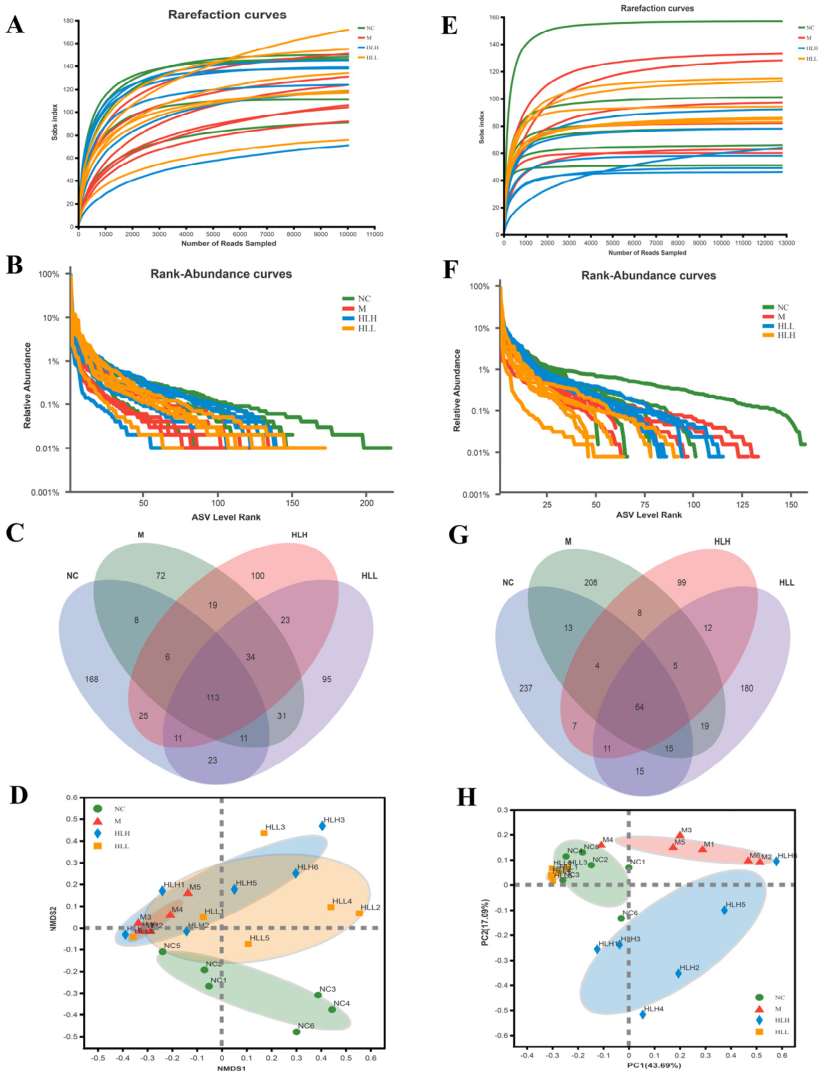

Figure S1. Bacterial and fungi community distribution. (A) Rarefaction curves based on Sobs index. (B) The grading curves, (C) Venn diagram analysis, (D) NMDS analysis based on Bray-Curtis algorithm of bacterial composition. (E) Rarefaction curves based on Sobs index. (F) The grading curves. (G) Venn diagram analysis. (H) PCA analysis of fungi composition.

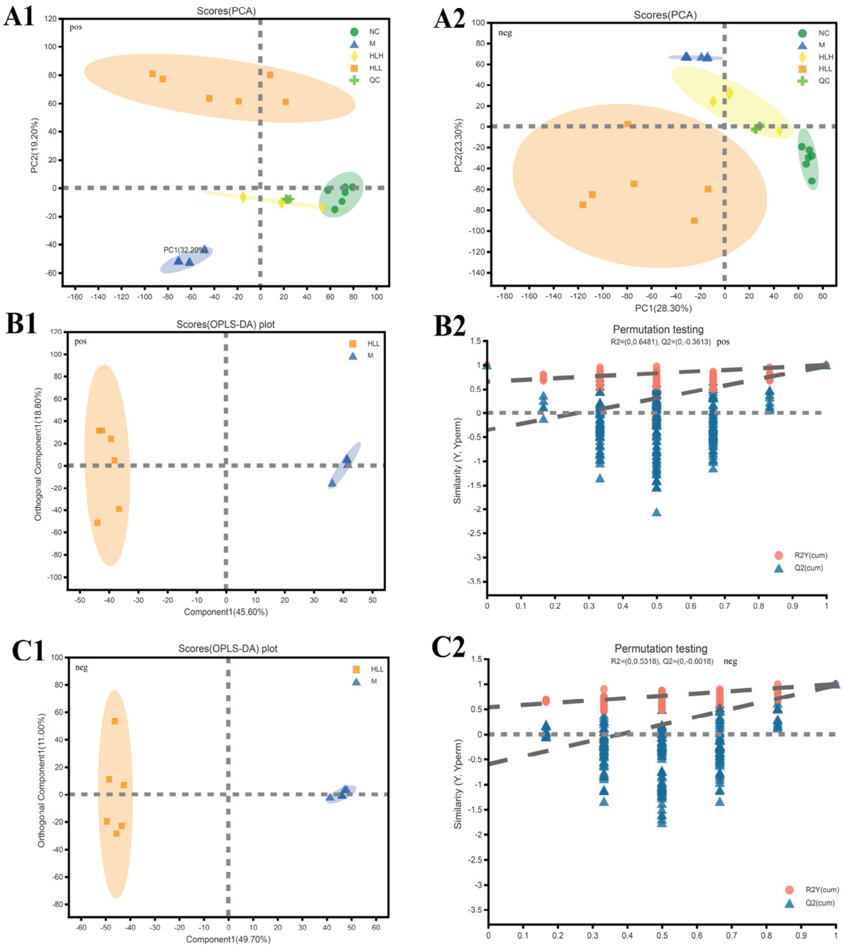

Figure S2. The effects of 10^2 spores/mL *E. amstelodami* H-1 (HLL) intervention on gut metabolism in colitis mice. PCA analysis of metabolome profiles (A1) positive and (A2) negative ion mode among the four groups of samples. OPLS-DA plot of metabolome profiles (B1, pos; C1, neg) and permutation testing (B2, pos; C2, neg) in HLL *vs* M (model).

Table S1. The standard scoring system of disease activity index (DAI).

**Score**	**Body weight loss**	**Feces status**	**Bloody stools**
0	no loss	normal	no blood (no color within 2 min)
1	0~10%	loose stool (not attached to the anus)	presence (Within 1~2 min, fuchsia)
2	10%~15%	loose stool (attach to the anus)	presence (Within 1 min, fuchsia)
3	15%~20%	diarrhea (liquid)	presence (Within 1 min, fuchsia)
4	>20%	severe diarrhea	gross blood (Instantly purplish blue)

Table S2. Sequences of Mouse Primers used for RT-PCR Analysis.

Primers	F	R
IL-1β	GCAACTGTTCCTGAACTCAACT	ATCTTTTGGGGTCCGTCAACT
IL-6	TAGTCCTTCCTACCCCAATTTCC	TTGGTCCTTAGCCACTCCTTC
IL-10	CTTACTGACTGGCATGAGGATCA	GCAGCTCTAGGAGCATGTGG
TNF-α	CCTGTAGCCCACGTCGTAG	GGGAGTAGACAAGGTACAACCC
TLR4	GCCTTTCAGGGAATTAAGCTCC	GATCAACCGATGGACGTGTAAA
p65	GCTTTGCAAACCTGGGAATA	TCCGCCTTCTGCTTGTAGAT
Nrf2	TAGATGACCATGAGTCGCTTGC	GCCAAACTTGCTCCATGTCC
COX-2	TTCAACACACTCTATCACTGGC	AGAAGCGTTTGCGGTACTCAT
iNOS	GGAGTGACGGCAAACATGACT	TCGATGCACAACTGGGTGAAC
ASC	CATCTTGTCTTGGCTGGTGGTCT	CGGACACGGACAGGATTGACA
Caspase-1	AATACAACCACTCGTACACGTC	AGCTCCAACCCTCGGAGAAA
NLRP3	TGTGAGAAGCAGGTTCTACTCT	TGTAGCGACTGTTGAGGTCCA
FXR	CTGCTTGTGATGCCACTTACA	TAGCACACGCTTCTCTCAGGT
PXR	TAGGGACCTGCCTATTGAGGA	CCGTTTCCGTGTCGAACATC
TGR5	CCTGGAACTCTGTTATCGCTCA	GCACTCGTAGACACCTTTGGG
GADPH	GCTCTGGCTCCTAGCACCAT	GCCACCGATCCACACAGAGT
